# Immunological Mechanisms and Effects of Bacterial Infections in Acute-on-Chronic Liver Failure

**DOI:** 10.3390/cells14100718

**Published:** 2025-05-15

**Authors:** Sumeng Li, Jing Liu, Jun Wu, Xin Zheng

**Affiliations:** 1 Department of Infectious Diseases, Union Hospital, Tongji Medical College, Huazhong University of Science and Technology, Wuhan 430030, China; d202281882@hust.edu.cn (S.L.); jingliu_19@126.com (J.L.); 2 Joint International Laboratory of Infection and Immunity, Huazhong University of Science and Technology, Wuhan 430022, China

**Keywords:** acute-on-chronic liver failure, bacterial infection, immune paralysis, immunomodulatory therapy

## Abstract

Acute-on-chronic liver failure (ACLF) is a severe clinical syndrome characterized by high morbidity and mortality rates. Bacterial infection is a frequent precipitating factor and complication in ACLF patients, significantly worsening patient outcomes. Elucidating the mechanisms underlying bacterial infections and their impact on ACLF pathophysiology is crucial for developing effective therapies to reduce infection rates and mortality. Current research highlights that immune suppression in ACLF increases susceptibility to bacterial infections, which in turn exacerbate immune dysfunction. However, a comprehensive review summarizing the emerging mechanisms underlying this immunosuppression is currently lacking. This review aims to provide an overview of the latest research, focusing on alterations in the immune responses of innate immune cells—including monocytes, macrophages, and neutrophils—as well as adaptive immune cells such as T and B lymphocytes during the onset and progression of bacterial infections in ACLF. In addition, recent advances in immunomodulatory therapies, including stem cell-based interventions, will also be discussed.

## 1. Introduction

Acute-on-chronic liver failure (ACLF) is characterized by an acute deterioration of liver function in a background of chronic liver disease, accompanied by organ failure and a high short-term mortality rate [1,2,3,4]. Previous studies showed that the 28-day mortality rate of ACLF patients ranges from 25.5% to 52.1% [5,6,7,8], which was significantly higher than that of non-ACLF patients. Bacterial infection is one of the most common complications in ACLF patients. It is reported that more than 60% of ACLF patients develop bacterial infections [8,9]. Moreover, bacterial infection is the most common extrahepatic precipitating factor in ACLF [10,11], accounting for 35–55% of ACLF patients [12,13,14,15,16]. The most prevalent type of bacterial infection in ACLF is spontaneous bacterial peritonitis (SBP), followed by urinary tract infections, pulmonary infections, and skin and soft tissue infections [17,18]. Gram-negative bacteria like *Escherichia coli* and *Klebsiella pneumoniae* are predominant pathogens [17,19,20].

Bacterial infections worsen prognosis, increasing mortality risk four-fold and reducing survival rates significantly. Bacterial infections can trigger progressive hepatic decompensation and a vicious cycle of recurrent infections. Without liver transplantation, the 1-year mortality rate of these patients exceeds 60% [11,21,22]. Studies have shown that among patients with end-stage liver disease (ESLD), the 3-, 6-, 12-, and 30-month survival rates in those diagnosed with bacterial infections are 50%, 46%, 41%, and 34%, respectively, which are markedly lower than the corresponding survival rates in patients without bacterial infections (83%, 77%, 71%, and 62%, respectively). And even after effective treatment and control of bacterial infections, patient survival remains adversely affected [23]. Another study reported that cirrhotic patients with bacterial infections have a fourfold increase in mortality risk [21]. Furthermore, SBP, the most common bacterial infection in ACLF, is closely associated with higher 30-day and 90-day mortality rates in ACLF patients [10].

Given the high incidence and mortality associated with bacterial infections in ACLF, it is essential to elucidate the mechanisms underlying bacterial infections and their role in the pathogenesis and progression of ACLF. This review focuses on the specific immune alterations in ACLF that contribute to increased susceptibility to bacterial infections, the reciprocal impact of infection on host immune responses, and provides an overview of current and emerging immunomodulatory therapeutic strategies.

## 2. Immune Paralysis in ACLF Increases the Risk of Bacterial Infections

Immune paralysis is considered the primary reason for the high susceptibility to bacterial infections in ACLF patients [24,25,26]. During the development and progression of ACLF, changes in the number and function of innate and adaptive immune cells impair the ability to eliminate pathogens, thereby increasing the risk of bacterial infections.

## 3. Innate Immune Dysfunction

Numerous studies worldwide have reported immune paralysis in decompensated cirrhosis and ACLF. As the first line of defense against invading pathogens, the innate immune system is the most affected during immune suppression. With the progression of cirrhosis, the integrity of the intestinal barrier is compromised, leading to increased bacterial translocation. Consequently, immune cells are persistently exposed to lipopolysaccharides (LPS) and other pathogen-associated molecular patterns (PAMPs), resulting in suppressed innate immune responses [27]. Among innate immune cells, monocytes, macrophages, and neutrophils undergo the most significant changes, contributing substantially to the increased incidence of bacterial infections.

## 4. Monocytes

As a critical component of the innate immune system, monocytes play an essential role in infection, tissue injury, and inflammation [28,29,30]. In ACLF patients, not only is the number of circulating monocytes increased [31], but their phenotype and function are also altered. Studies have shown that the expression of human leukocyte antigen-DR (HLA-DR) and genes related to immune response and cell activation are downregulated in classical monocytes of ACLF patients, while intracellular IL-10 levels of classical and intermediate monocytes were higher than those of healthy controls after exposure to LPS, indicating that monocytes of ACLF patients exhibit limited activation and functional suppression, leading to impaired pathogen clearance. [32]. Moreover, the number of CD14^+^ CD15^−^ CD11b^+^ HLA-DR^−^ monocytic myeloid-derived suppressor cells (M-MDSCs) is significantly increased in the peripheral blood of ACLF patients [24]. These cells represent a heterogeneous population with potent immunosuppressive activity and constitute a chief component of immunosuppressive networks [33]. M-MDSCs markedly inhibit T cell proliferation, exhibit reduced phagocytic capacity against bacteria, and show diminished production of cytokines upon stimulation [24]. The expansion of these cells is closely associated with elevated bacterial DNA levels in ACLF patients, significantly impairing antimicrobial immune responses [24]. In addition to M-MDSCs, studies have also identified an increased number of monocytes expressing Mer tyrosine kinase (MerTK) in the peripheral blood of ACLF patients [25,34]. MerTK is a transmembrane protein commonly expressed on the surface of monocytes/macrophages, dendritic cells, epithelial cells, and neuronal tissues, and serves as a key negative regulator of innate immune responses. Elevated MerTK expression reduces monocyte responsiveness to LPS and inhibits innate immune responses involved in pathogen clearance [25]. CD14^+^ HLA-DR^+^ AXL^+^ monocytes also expand progressively with the advancement of cirrhosis. These cells exhibit reduced cytokine secretion and diminished T cell activation after LPS stimulation, which is associated with a higher incidence of bacterial infections and increased 1-year mortality [35]. In contrast to the expansion of the aforementioned monocyte subsets, the number of CD52^high^ monocytes, a subset with potent phagocytic, cytokine-producing, and migratory capabilities essential for pathogen clearance and immune response initiation, is reduced in the peripheral blood of ACLF patients [36]. In sepsis, high CD52 expression is closely associated with favorable outcomes [37]. However, in ACLF patients, CD52 expression is downregulated, while soluble CD52 (sCD52), which exerts broad immunosuppressive effects, is increased [38]. Furthermore, ACLF patients exhibit increased CD163 expression on monocytes, reduced phagocytic and oxidative burst capacity, and impaired secretion of pro-inflammatory cytokines upon activation [24,39,40]. These quantitative and functional alterations in monocytes predispose ACLF patients to recurrent bacterial infections, organ failure, and even death [8]. Decreased phagocytosis of monocytes, the expression of immunosuppressive phenotypes, and increased frequency of suppressor cells lead to decreased ability to clear bacteria and increased risk of bacterial infection.

## 5. Macrophages

Similarly to monocytes, macrophages also showed functional inhibition. Macrophages are the primary phagocytes in the liver [41], playing a pivotal role in hepatic antimicrobial defense [42]. Macrophage-mediated inflammatory responses are indispensable in the pathogenesis and progression of ACLF [43], and during this process, both the number and function of macrophages undergo significant alterations. China L and colleagues stimulated healthy macrophages with plasma from ACLF patients and observed a reduced capacity for TNF secretion compared to healthy controls, indicating macrophage dysfunction in ACLF patients [44]. Studies have shown that the expression of CD14, a receptor for LPS, is downregulated on macrophages in SBP patients, which reduces their phagocytic capacity [45,46]. Additionally, serum levels of prostaglandin E2 (PGE2), an inhibitory molecule that diminishes macrophage bactericidal activity, are elevated in ACLF patients [39]. RNA sequencing of hepatic non-parenchymal cells (NPCs) in ACLF patients revealed Kupffer cell depletion and enrichment of immunosuppressive TREM2^+^ macrophages derived from monocytes, contributing to the formation of an immunosuppressive microenvironment [47]. Zhang Y and colleagues reported that in the liver tissues of HBV-ACLF patients, the proportion of CD68^+^ HLA-DR^+^ macrophages was elevated, along with increased levels of IL-1β and TGF-β1, whereas the proportion of CD206^+^ CD163^+^ macrophages was decreased, accompanied by reduced IL-10 expression [48]. These findings indicate that intrahepatic macrophages in HBV-ACLF exhibit a pro-inflammatory phenotype, where excessive inflammation exacerbates disease progression and further increases the risk of infection. Furthermore, compared to patients with stable cirrhosis, ACLF patients show a significant increase in the number of macrophages expressing MerTK in the liver, which suppresses macrophage immune responses against invading pathogens [25]. The quantitative and functional alterations of macrophages impair the host’s ability to clear pathogenic microorganisms, compromising normal antibacterial immune responses and further increasing the risk of bacterial infections in patients with ACLF.

## 6. Neutrophils

Alongside monocytes and macrophages, neutrophils are also one of the main innate immune cells that exhibit functional inhibition. Neutrophils are the most abundant innate immune cells in the human body, exerting their functions through phagocytosis, degranulation, and the formation of neutrophil extracellular traps (NETs) [49,50], forming the first line of defense against bacterial infections and tissue damage. The number of circulating neutrophils is significantly increased in ACLF patients, which serves as an independent risk factor for disease severity and poor prognosis [51,52,53]. However, despite the increased neutrophil count, their phagocytic capacity is severely impaired, which is the most notable functional abnormality observed in neutrophils from ACLF patients [51]. In contrast to the decline in phagocytic ability, the capacity of neutrophils to form NETs is enhanced in ACLF patients, potentially driven by high-mobility group box 1 (HMGB1) released during hepatocyte pyroptosis, which promotes NET formation [54]. Increased NET formation is particularly evident in ACLF patients with poor prognosis, possibly due to excessive NETs leading to immune-mediated tissue damage [55,56]. Although NETs play a critical role in eliminating bacterial, fungal, and viral pathogens [57,58,59,60,61,62], excessive NET formation can have deleterious effects. On the one hand, excessive NETs promote thrombosis, exacerbating the bleeding tendency in ACLF patients, while on the other hand, they induce apoptosis, leading to tissue damage and inflammation. Subsequently, the release of death-associated molecular patterns (DAMPs) from dying cells further activates neutrophils, perpetuating a vicious cycle of NET formation and inflammatory damage [63,64]. Moreover, neutrophil migration capacity is impaired in patients with ACLF [65], and in some patients, neutrophils fail to migrate even under resting or stimulated conditions, placing them at a higher risk of developing sepsis or death [66]. The quantitative and functional defects of neutrophils contribute to the increased susceptibility to bacterial infections and the higher short-term mortality observed in ACLF patients [67]. The diminished phagocytic capacity of neutrophils, along with their tendency to trigger excessive inflammatory responses, contributes to the onset and progression of bacterial infections.

## 7. Adaptive Immune Suppression

In addition to impaired innate immune responses, adaptive immune responses are also altered in ACLF patients. These alterations, although perhaps later than the innate immune response, also further promote bacterial infection.

## 8. T Cells

Studies have reported a significant reduction in the number of peripheral CD4^+^ and CD8^+^ T cells in ACLF patients [68,69], with the decrease primarily affecting conventional CD4^+^ T cells (CD4^+^CD25^−^/^+^ T cells), resulting in an increased frequency of regulatory T cells (Tregs) [70]. The expanded Treg population further suppresses the proliferation of autologous CD4^+^CD25^−^ T cells [70]. An increase in Treg frequency is associated with disease progression and severity in ACLF patients [69,71]. In addition to numerical decline, CD4^+^ T cells exhibit limited activation. Studies have shown that CD4^+^ T cells from HBV-ACLF patients exhibit increased expression of B- and T-lymphocyte attenuator (BTLA), an immunoglobulin domain-containing protein involved in maintaining peripheral tolerance and limiting immunopathological damage during immune responses [72]. BTLA suppresses CD4^+^ T cell activation, proliferation, and cytokine production, and promotes CD4^+^ T cell apoptosis. Blocking BTLA expression using antibodies in a mouse model of ACLF reduced bacterial load and decreased mortality [9]. Similarly, CD8^+^ T cells in ACLF patients are not only reduced in number but also display diminished T cell receptor (TCR) repertoire diversity [73]. A diverse TCR repertoire is essential for recognizing a wide range of antigens. Upon bacterial infection, CD8^+^ T cells with TCRs specific for the corresponding pathogen antigens are activated, proliferate, differentiate, and migrate to the site of infection to eliminate the pathogen [74,75]. Therefore, a reduction in TCR diversity inevitably compromises the ability of CD8^+^ T cells to mount an effective response against bacterial pathogens. Furthermore, the number of activated CD8^+^ T cells is reduced in ACLF patients [68], while cytotoxic T lymphocyte (CTL) activity is paradoxically enhanced [76]. Additionally, there is an expansion of inhibitory CD8^+^ T cells expressing high levels of Tim-3, which correlates with an increased risk of infection and disease progression [77]. The reduced numbers of CD4^+^ and CD8^+^ T cells, along with the upregulation of inhibitory molecules and decreased TCR diversity in CD8^+^ T cells, impair the pathogen-specific immune response and facilitate the development of bacterial infections.

## 9. B Cells

Research on B cells in ACLF remains relatively limited, but existing studies suggest that both the number and function of B cells are altered. Compared to patients with cirrhosis and healthy controls, ACLF patients show a decrease in circulating and hepatic naïve B cells, accompanied by an increased proportion of memory B cells and plasma cells [78]. These changes in B cell populations are closely associated with disease progression and severity in ACLF patients [78]. Although current evidence does not directly link these B cell changes to bacterial infections in ACLF, it is speculated that worsening disease may further elevate the risk of infection.

**Figure 1:**
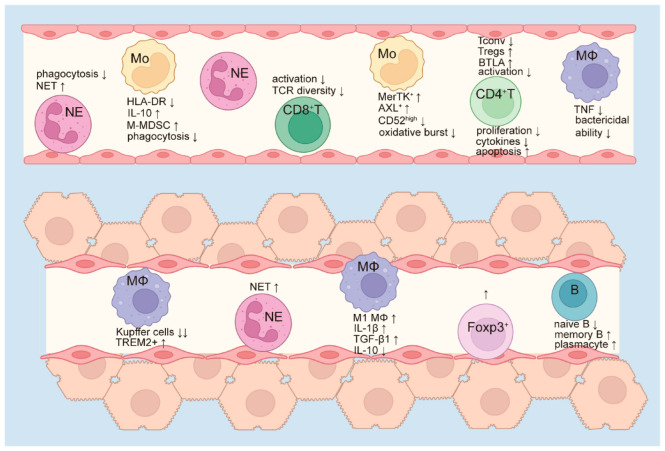
Immune paralysis in ACLF. As ACLF progresses, circulating neutrophils and monocytes accumulate, but their functions are markedly altered. Neutrophils exhibit impaired phagocytic capacity but enhanced formation of neutrophil extracellular traps (NETs), which may trigger a more intense inflammatory response. Monocytes demonstrate reduced phagocytosis and diminished oxidative burst, accompanied by increased secretion of IL-10. The proportions of monocytic myeloid-derived suppressor cells (M-MDSCs), MerTK^+^, and AXL^+^ monocytes are elevated, while CD52^high^ monocytes are decreased. Circulating macrophages secrete lower levels of TNF and exhibit reduced bactericidal activity. Meanwhile, activated CD8^+^ T cells are reduced, along with a reduction in T cell receptor (TCR) diversity. CD4^+^ T cells show a decline in conventional subsets, with a relative increase in regulatory T cells (Tregs), elevated inhibitory molecule BTLA expression, and reduced activation and cytokine production. Proliferation of CD4^+^ T cells is diminished, while apoptosis is increased. The innate and adaptive immunity in peripheral blood were suppressed. In the liver, the number of resident Kupffer cells decreases, whereas TREM2^+^ macrophages become more abundant. Macrophages tend to polarize toward the pro-inflammatory M1 phenotype, characterized by increased secretion of IL-1β and TGF-β1 and decreased production of IL-10. Hepatic neutrophil infiltration is also enhanced, with increased NET formation. Additionally, the numbers of Foxp3^+^ cells, memory B cells, and plasma cells increase, while naïve B cells are reduced. Collectively, these alterations reflect a paradoxical immune state in which pro-inflammatory responses are activated but immune cell functions are impaired, thereby compromising the host’s ability to effectively clear pathogens. ↑ indicates an increase in frequency, quantity or expression level. ↓ represents a decrease in the frequency, quantity or expression level. NE, neutrophil. Mo, monocyte. MΦ, macrophage. B, B cells. NET, neutrophil extracellular trap. HLA-DR, human leukocyte antigen DR. M-MDSC, monocytic myeloid-derived suppressor cells. MerTK, Mer tyrosine kinase. BTLA, B- and T-lymphocyte attenuator. TREM 2, Triggering Receptor Expressed on Myeloid Cells 2. M1 MΦ, M1 macrophages.

In summary, innate immune suppression typically occurs earlier than adaptive immune suppression and affects a broader spectrum of immune cells. As the disease worsens, the numbers of circulating neutrophils and monocytes increase, but their phagocytic capacity declines, contributing instead to an exaggerated inflammatory response. Concurrently, there is an expansion of immunosuppressive monocytic subpopulations and upregulation of inhibitory molecules. The bactericidal activity of circulating macrophages is also diminished, leading to a compromised innate immune response and reduced ability to clear pathogens. In parallel, activated CD8^+^ T cell numbers decline along with reduced T cell receptor (TCR) diversity, weakening the adaptive immune response to eliminate bacteria. CD4^+^ T cells undergo increased apoptosis and a decrease in overall frequency, resulting in a relative increase in Treg proportions. Within the liver, there is a paradoxical state of heightened pro-inflammatory signaling alongside impaired immune cell function, leading to insufficient clearance of invading pathogens (Figure 1). The extensive alterations in both innate and adaptive immune responses in ACLF patients significantly increase their susceptibility to bacterial infections, which further exacerbates disease progression and clinical deterioration.

## 10. Impact of Bacterial Infections on ACLF Immunity

The series of immune alterations observed in ACLF patients increases their susceptibility to bacterial infections, which, in turn, can significantly impact the immune response and prognosis of these patients.

Studies have reported that higher bacterial DNA concentrations in the ascitic fluid are associated with the increased production of pro-inflammatory cytokines and reduced HLA-DR expression on CD14^+^ peritoneal macrophages [79,80,81]. Bacteria or bacterial products such as LPS stimulate the production of inflammatory cytokines, including TNF-α, IL-6, calprotectin, and macrophage inflammatory protein-1β (MIP-1β), which further promote the recruitment of neutrophils and monocytes [82,83,84]. Although monocytes continue to accumulate following LPS stimulation, Yadav P. et al. reported that in ACLF patients with concurrent sepsis, monocytes exhibit reduced HLA-DR expression and increased expression of PD-L1 and Tim-3 [85], indicating further functional suppression of monocytes and a diminished capacity to clear pathogens. Previous studies have shown that elevated bacterial DNA levels in ACLF patients correlate with the frequency of monocytic myeloid-derived suppressor cells (M-MDSCs) [86]. Repeated stimulation of Toll-like receptor 2 (TLR-2) and/or TLR-4 induces the expansion of M-MDSC-like suppressive cell populations [24]. Increased M-MDSC frequency further exacerbates immune suppression. Additionally, bacterial infections engage PAMPs with pattern recognition receptors (PRRs), leading to functional alterations in neutrophils in ACLF patients [51].

Beyond innate immune cells, bacterial infections also significantly affect the adaptive immune response. Studies have shown that LPS-induced IL-6 and TNF-α may upregulate BTLA expression on CD4^+^ T cells via the STAT3 and NF-κB signaling pathways, further inhibiting CD4^+^ T cell function [9].

**Figure 2:**
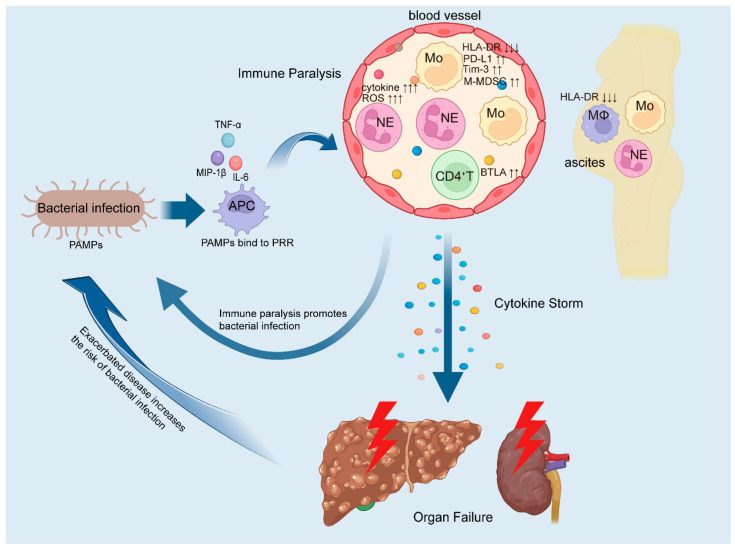
Bacterial infection drives a fatal cycle of infection-immune paralysis-reinfection-organ failure. Bacterial components, as pathogen-associated molecular patterns (PAMPs), bind to pattern recognition receptors (PRRs) on antigen-presenting cells (APCs), thereby stimulating APCs to release pro-inflammatory cytokines such as TNF-α, IL-6, and MIP-1β. These cytokines further promote the recruitment of circulating neutrophils and monocytes, leading to the release of inflammatory cytokines and reactive oxygen species (ROS) by neutrophils, ultimately triggering a hyperinflammatory state. Meanwhile, Compared with uninfected ACLF patients, the expression of activation marker HLA-DR on monocytes was further decreased, while the expression of inhibitory molecules PD-L1 and Tim-3 was up-regulated, along with an increased frequency of immunosuppressive monocytic myeloid-derived suppressor cells (M-MDSCs). In parallel, CD4^+^ T cells exhibit elevated expression of inhibitory molecule BTLA. Additionally, HLA-DR expression on macrophages in ascitic fluid is also further reduced. These changes indicated that both innate and adaptive immune suppression were further aggravated after bacterial infection. This exacerbated immune dysfunction in turn heightens the risk of bacterial infections. At the same time, the imbalance between pro-inflammatory and anti-inflammatory responses leads to a cytokine storm, contributing to organ failure and deterioration. Disease progression further increases susceptibility to bacterial infection, creating a vicious cycle of infection, immune paralysis, reinfection, and organ failure. ↑ indicates an increase in frequency, quantity or expression level. ↓ represents a decrease in the frequency, quantity or expression level. MIP-1β, macrophage inflammatory protein-1β. NE, neutrophil. Mo, monocyte. MΦ, macrophage. ROS, reactive oxygen species. HLA-DR, human leukocyte antigen DR. PD-L1, programmed death ligand 1. Tim 3, T cell immunoglobulin and mucin domain-3. M-MDSCs, immunosuppressive monocytic myeloid-derived suppressor cells. BTLA, B- and T-lymphocyte attenuator.

As the disease progresses, ACLF patients develop immune paralysis, leading to a marked increase in bacterial infection rates. These invading bacteria bind to PRRs, triggering antigen-presenting cells (APCs) to release pro-inflammatory cytokines. This, in turn, induces excessive activation of circulating neutrophils and upregulates the expression of inhibitory molecules on monocytes and CD4^+^ T cells. Additionally, the number of immunosuppressive monocyte subsets rises, while activation markers on macrophages in ascitic fluid are downregulated. These changes suggest that bacterial infections further exacerbate immune dysfunction [16,87,88]. This worsening immune dysregulation contributes to a cytokine storm, leading to multi-organ dysfunction—particularly of the liver and kidneys—and accelerates disease progression. Consequently, the host enters a vicious cycle of infection, clinical deterioration, reinfection, and further decompensation, ultimately culminating in multi-organ failure and potentially death. This vicious cycle is illustrated in Figure 2.

Due to the limited availability of fresh liver tissue samples, studies on the dynamic changes within the intrahepatic immune microenvironment are scarce. Most current investigations into the impact of bacterial infections in ACLF focus on clinical outcomes based on cohort data, with little insight into how such infections specifically alter both systemic and liver-localized immune responses and how these changes influence disease progression and prognosis. To address these gaps, future research should integrate analyses of both fresh liver tissue and peripheral blood to provide a more comprehensive picture of the immune landscape in ACLF. There is also a pressing need for more robust and representative animal models of liver failure to elucidate the dynamic interplay between bacterial infection, immune dysregulation, and disease progression. In addition, advances in organoid technologies may soon allow the widespread use of human liver organoids derived from primary hepatic tissue or hepatocytes. These platforms hold great promise for dissecting the mechanisms underlying immune paralysis in ACLF, particularly the ways in which bacterial infections exacerbate immune suppression and drive clinical deterioration.

## 11. Immunomodulatory Therapies

Due to the high incidence and mortality rates of ACLF, there is an urgent need for safe and effective therapeutic approaches to meet clinical demands. Timely empirical anti-infective treatment can significantly reduce the 28-day mortality rate in ACLF patients [19,89], which is key to preventing and controlling bacterial infections in this patient population. In addition to antibiotic therapy, immunomodulatory treatment is garnering increasing attention.

**Macrophage modulation:** Given the critical role of macrophages in hepatic antibacterial immunity and inflammation, modulating macrophage function in ACLF has become a focal point of immunomodulatory therapy. Bacterial components such as LPS, a PAMP, bind to TLR4 and activate macrophages [90]. TLR4 inhibitors (TAK-242 or Serelaxin) can block the interaction between PAMPs and TLR4, thus inhibiting macrophage activation [84]. Chemokines CCL2 and CCL5, along with their respective receptors CCR2 and CCR5, constitute a major chemotactic axis that facilitates immune cell migration to the liver [91,92]. The use of CCR2 and CCR5 antagonists can disrupt this chemotactic axis, preventing monocyte infiltration into the liver and reducing the number of monocyte-derived macrophages [92]. Moreover, certain substances such as taurine [93], unsaturated fatty acids [94], and salbutamol [95] have been shown to inhibit macrophage polarization toward the pro-inflammatory M1 phenotype. Conversely, mesenchymal stem cells (MSCs) can promote macrophage polarization toward the anti-inflammatory M2 phenotype through the Mertk/JAK1/STAT6 signaling pathway in ACLF mouse models [96]. These agents help mitigate inflammatory responses and reduce the risk of bacterial infections. Additionally, the infusion of 20% human albumin can lower plasma PGE2 levels, thereby restoring the bactericidal capacity of macrophages and monocytes [44].

**Neutrophil regulation:** As previously mentioned, neutrophil dysfunction and excessive NET formation also increase the risk of bacterial infections and accelerate disease progression. Studies have demonstrated that protein arginine deiminase 4 (PAD4) inhibitors can inhibit neutrophil NET formation [97], thereby alleviating NET-induced inflammation.

**T cell restoration:** There are few reports on T cell immunomodulatory therapy in ACLF. The use of anti-BTLA antibodies in ACLF mouse models has been shown to reduce *Klebsiella pneumoniae* load and improve survival rates [9].

**Mesenchymal stem cell therapy:** Mesenchymal stem cell (MSC) therapy has been reported to significantly improve short-term survival in patients with acute-on-chronic liver failure (ACLF) [98,99]. In addition to alleviating liver injury by promoting hepatocyte regeneration and inhibiting hepatocyte apoptosis [100,101,102], MSCs also exert immunomodulatory effects. They can migrate to sites of inflammation and secrete various soluble factors that promote the proliferation and functional regulation of multiple immune cell types, including regulatory T cells (Tregs), regulatory B cells, and M2-polarized macrophages [103], while simultaneously suppressing neutrophil recruitment to sites of injury [104]. These actions collectively induce a robust anti-inflammatory response [105], thereby further alleviating liver damage. MSC therapy has been shown to delay disease progression and reduce the risk of severe bacterial infections [99]. However, data regarding the long-term impact of MSC therapy on the prognosis of ACLF patients are currently lacking, and whether MSCs can improve long-term survival remains to be confirmed by further prospective clinical studies. Moreover, the potential tumorigenic risk associated with MSC therapy warrants particular caution.

Although research on immunomodulatory therapies is expanding, the translation of these findings into effective treatments for ACLF patients remains limited. Clinicians and researchers must recognize that the immune response is a double-edged sword: insufficient immune activation may increase susceptibility to bacterial infections and accelerate disease progression, while excessive immune responses may provoke autoimmune damage and exacerbate the condition. Therefore, the timing, choice of immunomodulatory agents, and intensity of immune modulation require careful evaluation. Further studies using appropriate animal models and clinical trials are essential to identify safe and effective immunomodulatory agents, optimal dosing regimens, and appropriate treatment durations for ACLF patients.

## 12. Conclusions

In addition to exacerbating immune suppression and inflammatory responses, bacterial infections in ACLF patients often go undetected due to the inherently low positive rates of clinical microbial cultures, resulting in delays in antibiotic treatment. Moreover, severe bacterial infections may prevent patients from receiving timely liver transplantation, further worsening their prognosis. These factors collectively contribute to the severity of illness and increased mortality in ACLF patients with bacterial infections. Bacterial infections accelerate the onset and progression of ACLF, leading to high morbidity and mortality, and posing significant challenges for clinicians. A comprehensive understanding of how immune suppression in ACLF facilitates bacterial infections—and, conversely, how these infections further impair immune responses—can aid clinicians in promptly assessing infection status, initiating timely antimicrobial therapy, and introducing immunomodulatory interventions at appropriate stages.

However, it is important to acknowledge that while substantial research has been conducted on innate immune alterations in the peripheral blood of ACLF patients, studies focusing on changes in adaptive immunity—particularly B cell responses—remain limited.

With a deeper mechanistic understanding, there is hope that novel, safe, and effective immunomodulatory therapies—including targeted monoclonal antibodies—can be developed for clinical application. When combined with adequate antimicrobial treatment, such approaches may help restore immune function, improve clinical outcomes, and ultimately reduce the rates of disability and mortality associated with ACLF.

## Data Availability

No new data were created or analyzed in this study.
